# The Physiology of Digestion, Considered with Relation to the Principles of Dietetics

**Published:** 1836-10

**Authors:** 


### Art. IV.

*The Physiology of Digestion, considered with Relation to the Principles of Dietetics.* By Andrew Combe, m.d. &c.—*Edinburgh*, 1836. 8vo. pp. 332.

For the present volume from the pen of Dr. Combe we were prepared by an announcement in the third edition of his "Principles of Physiology applied to the Preservation of Health," in which he stated that, in compliance with suggestions that had been frequently made to him, it was his intention to give an account of digestion, nutrition, the circulation, &c., in a future work. The great popularity of the former publication will doubtless have already given a wide circulation to the present production; although the author will not, we are sure, deceive himself with the expectation that the public, who received his first volume with such great favour, will be found industrious enough to peruse a second and a third volume, however worthy of their perusal, with equal diligence. Those, in fact, who are most in need of the instruction such works convey are not the most likely persons to be very persevering readers. One instructive book would be received with submission by many whose indolence will deter them from the study of a series of volumes. The usefulness, therefore, of this treatise on the Physiology of Digestion will be much more limited, we apprehend, than that of the Principles of Physiology, merely because it comes in the shape of a second book, and a whole book upon one subject.

Yet, of all subjects which interest the health of mankind, and of all subjects upon which mankind commit the greatest number of daily errors, that of Digestion and of Dietetics is the most remarkable. Considering how many persons there are with whom eating and drinking form the most interesting events of the day; reflecting, too, on the care taken to refine the arts which give greater enjoyment to the comforts of the table; and bearing in mind the almost universal outcry concerning indigestion, and the absolute necessity of eating and drinking; it is really humiliating to reflect upon the ignorance of people in general respecting the food proper for themselves and for their children, and for persons of different habits and different ages, and in different climates and seasons, and in different states of health. If the utmost ignorance is a state favorable to the reception of knowledge, the public are certainly in a most appropriate condition to receive Dr. Combe's book.

Although his work is chiefly intended for the general reader, we notice it in conformity to the principle by which we were governed in reviewing several works on Public and Private Hygiène, in our second Number. We are persuaded that there are many and grievous ills arising from impaired digestion, which, when once incurred, are most difficult of relief, if indeed they admit of perfect relief from medicine. In the treatment of all such maladies, too, the cure must be partly dietetic; and we well know that too many medical men are destitute of steady dietetic principles, embarrassed when required to lay down dietetic rules, and prone to rush into dogmatic absurdities on this subject with regard to patients for whom nothing can be too absurd, provided it be authoritatively delivered. Thus it is that the plain practitioner, who has dismissed his patient in all good faith to seek health from change of air, finds, on the patient's return from his travels, that there is much to be learned concerning diet and regimen; that it is perfectly proper, for instance, in chronic organic diseases, to eat portions of animal food, highly seasoned, two or three times a day, and to take as often two or three glasses of sherry; but, at the same time, quite essential to shun the smallest fragment of pudding and every description of vegetable, as if to eat it were certain death. If sherry has been prescribed to one patient, another has been assured that his life depends upon brandy: to one, champagne has been recommended as the balm of life, and to another beer, as the only hope of earthly salvation; whilst perhaps both have been solemnly warned from the enormity of tea, but allowed coffee and liqueurs, or (by a more rigid sentence) condemned to sip cold water at tea-time, with the addition of a precise number of spoonfuls of salt. By this kind of system, if system it can be called, sometimes a bon-vivant may become, to be sure, an involuntary member of the society of the temperate; but many a lady, on the other hand, is introduced to the consolation of dram-drinking; and many a patient, who might have lingered through a few months, or even years, of valetudinarian life, is despatched in a few weeks. And all this outrageous dictation is submitted to because people, otherwise tolerably educated, are grossly ignorant of physiology, and because medical practitioners pay too little attention to the regulation of the diet of their patients. Surely, then, both patients and practitioners may with much advantage be advised to read Dr. Combe's book; the practitioner to supply himself with plain rules founded on plain reasons, and the patient to qualify himself to understand those reasons, so as to ensure his obedience to rational regulations, and his contempt for the arbitrary mandates of the presumptuous and the unscientific.

Dwelling briefly on the obvious facts that animals intended for locomotion, not being capable of receiving nourishment from fixed roots, are provided with a receptacle or stomach, from the supplies taken into which the whole body is to receive nutritious matter; and that the keeping up of this supply is not left dependent on the will, but instigated by the resistless force of hunger and thirst; Dr. Combe proceeds, in his second chapter, to treat of these appetites, illustrating those points which he wishes to impress on his readers with his usual felicity. The experiments of Brachet, to show the degree of dependence of the sense of hunger on the connexion of the stomach with the brain, are thus mentioned:

"Brachet starved a dog for twenty-four hours, till it became ravenously hungry, after which he divided the nerves which convey to the brain a sense of the condition of the stomach. He then placed food within its reach, but the animal, which a moment before was impatient to be fed, went and lay quietly down, as if hunger had never been experienced. When meat was brought close to it, it began to eat; and, apparently from having no longer any consciousness of the state of its stomach—whether it was full or empty,—it continued to eat till both it and the gullet were inordinately distended. In this, however, the dog was evidently impelled solely by the *gratification of the sense of taste;* for on removing the food to the distance of even a few inches, it looked on with indifference, and made no attempt either to follow the dish or to prevent its removal.

"Precisely similar results ensued when the nervous communication between the stomach and the brain was arrested by the administration of narcotics. A dog suffering from hunger turned listlessly from its food when a few grains of opium were introduced into its stomach. It may be said that such a result is owing to the drug being absorbed and carried to the brain through the ordinary medium of the circulation: but Brachet has proved that this is not the case, and that the influence is primarily exerted upon the nerves. To establish this point, two dogs of the same size were selected. In one the nerves of communication were left untouched, and in the other they were divided. Six grains of opium were then given to each at the same moment. The sound dog began *immediately* to feel the effects of the opium and became stupid, while the other continued lying at the fire-side for a long time, without any unusual appearance except a little difficulty of breathing. In like manner, when the experiment was repeated with that powerful poison *nux vomica,* upon two dogs similarly circumstanced, the sound one fell *instantly* into convulsions, while the other continued for a long time as if nothing had happened.

"These results demonstrate, beyond the possibility of doubt, the necessity of a free nervous communication between the stomach and the brain, for enabling us to experience the sensation of hunger. The connexion between the two organs is indeed more widely recognized in practice than it is in theory; for it is a very common custom with the Turks to use opium for abating the pangs of hunger when food is not to be had, and sailors habitually use tobacco for the same purpose. Both substances act exclusively on the nervous system." (P. 14.)

The relation thus shown to exist between the stomach and the brain is then spoken of by Dr. Combe, with reference to certain facts sufficiently well known,—as the spoiling of the appetite by bad news, and the insensibility to its claims in times of earnest attention or intense study. It is the peculiar merit of the author to connect facts, most of which are familiarly known to medical readers, with observations useful to those unacquainted with medical subjects; and he takes much, but not at all too much, pains to convince the reader of the necessity of proportioning the supply of food to the waste of the body, by showing that, whenever the waste is greatest, the desire for food is proportionally increased. He justly remarks, that forgetfulness of this piece of knowledge is a frequent source of indigestion and ill health. There is not a word of the following passage that is not true, and we extract it on account of its pointing to a very common cause of ill health, against which medicine is often opposed in vain.

"There are numerous persons, especially in towns and among females, who, having their time and employments entirely at their own disposal, carefully avoid every thing which requires an effort of mind or body, and pass their lives in a state of inaction entirely incompatible with the healthy performance of the various animal functions. Having no bodily exertion to excite waste, promote circulation, or stimulate nutrition, they experience little keenness of appetite, have weak powers of digestion, and require but a limited supply of food. If, while inactive, and spending little, such persons could be contented to follow nature so far as not to provoke appetite by stimulants and cookery, and to eat and drink only in proportion to the wants of the system, they would fare comparatively well. But having no imperative occupation, and no enjoyment from active and useful exertion, their time hangs heavily on their hands, and they are apt to have recourse to eating as the only avenue to pleasure still open to them; and, forgetful or ignorant of the relation subsisting between waste and nutrition, they endeavour to renew, in the present indulgence of appetite, the real enjoyment which its legitimate gratification afforded under different circumstances. Pursuing the pleasures of the table with the same ardour as before, they eat and drink freely and abundantly, and, instead of trying to acquire a healthy desire for food and increased powers of digestion by exercise, they resort to tonics, spices, wines, and other stimuli, which certainly excite for the moment, but eventually aggravate the mischief by obscuring its progress and extent. The natural result of this mode of proceeding is, that the stomach becomes oppressed by excess of exertion—healthy appetite gives way, and morbid craving takes its place—sickness, headach, and bilious attacks, become frequent—the bowels are habitually disordered, the feet cold, and the circulation irregular—and a state of bodily weakness and mental irritability is induced, which constitutes a heavy penalty for the previous indulgence. So far, however, is the true cause of all these phenomena from being perceived even then, that a cure is sought, not in a better regulated diet and regimen, but from bitters to strengthen the stomach, laxatives to carry off the redundant materials from the system, wine to overcome the sense of sinking, and heavy lunches to satisfy the morbid craving which they only silence for a little. Some, of course, suffer in a greater and others in a less degree, according to peculiarities of constitution, mode of life, and extent of indulgence; but daily experience will testify, that, in its main features, the foregoing description is not overcharged, and that victims to such dietetic errors are to be met with in every class of society." (P. 23.)

The extent to which errors of this kind are practised is almost incredible; insomuch that, in nine-tenths of mankind, the first loss of health is to be attributed to a disproportion between food and exercise. The poor labourer suffers from a disproportion over which he has no control; his toils are severe, and his food is scanty and not of the most nourishing kind; but, although for this he incurs premature old age, his sufferings of mind and body are not so great as the afflictions which assail those who are in what are called comfortable circumstances. Men are disposed to congratulate themselves when they can indulge in an easy chair after dinner, heedless of the calls of business which once they so alertly attended to. The tradesman leaves the cares of his shop to those whom he can trust; the merchant flies far away from his office, to dine and to doze; the lawyer, once so energetic, can no longer be spoken with at ungenteel hours; and even the physician cheers his younger brethren by resisting the commands of rich and powerful patients, unless they choose to be ill in the morning. But for this abandonment of duty for ease, sure retribution follows. The waste becomes less, from inaction of body and of mind; the food taken is usually richer and more abundant than it was in days of struggling industry; and the stomach loses some of its power, and digestion and assimilation become imperfect; and the skin, or kidneys, become over-exerted, and gout or gravel, or rheumatic pains, or the various troubles of plethora, break in upon the dream of an idle and contented retirement. Medicine alone can neither avert nor remedy evils thus arising; and the precepts of medical men will be best enforced, best remembered, and best followed, if the reason is also stated, and the patient is convinced that he must either be more active, or, if age or other circumstances forbid that, must diminish the copiousness and richness of the material supply.

We are never more sensible of the degradation into which rational creatures may fall, than when we see the wealthy and luxurious resorting to watering places for no other object but to enable themselves to pursue a course of gluttonous excess with impunity. Each morning, to the sound of music, may be beheld the pampered and the selfish of the higher and middle classes, endeavouring to make a composition with nature for the previous day's excess, and for neglect of bodily exercise and mental activity, by drinking huge goblets of nauseous saline water. Crowds of persons of both sexes seem to take a pride in exhibiting themselves thus taking purgatives; and the disgusting custom of walking about afterward, (in lovely gardens, perhaps, where every object is noble but themselves, who are merely meditating on the salts they have swallowed,) is continued to the very verge of indecency. The great object for which the waters are commonly drunk being obtained, (an object much inferior, in our opinion, to what the saline waters are really capable of effecting, by a gradual alteration of the circulating fluids,) the invalids repair to their respective hotels, and, by the aid of many stimulants and provocatives, contrive to eat and drink during the day, after a fashion equally unsalutary and inelegant. If an American writer chose to return the poor spite of some of our English travellers in the United States, he would find abundant materials at the tables d'hôte and in the public rooms of any fashionable spa; and, without departing in the least particular from the truth, might present a picture of our unhappy countrymen and women, in which excess, idleness, and apathy as regards all good and elevated pursuits, furnish the principal colouring. He might also, if a medical observer, hold up to admiration the people of the comfortable classes of an enlightened country leading a life the most conducive to mental and bodily disease, out of pure anxiety to enjoy the utmost possible indulgence, and to suffer the least possible uneasiness. With such classes of the people, the medical teacher is certainly very much wanted.

The value of good teeth, as respects good digestion, is not sufficiently regarded. A desire to maintain the beautiful appearance of the teeth, which might supply the want of knowledge of their great importance as auxiliaries of the stomach, so often leads to the habitual application of tooth-powders capable of destroying them, that Dr. Combe very properly devotes a few pages to the subject of their preservation. Our private opinion is, that there are few tooth-powders that are entirely harmless; many are known to be directly prejudicial by their acid properties, and some we suspect to be very injurious by their coarse mechanical operation. The important object is to keep the surface of the teeth clean, which may be effected by using a soft tooth-brush dipped in tepid water after every meal, and also at night, always avoiding too much violence. But it is in vain to strive, by any applications, to keep the teeth in health, unless a rational system of diet be also observed. A disordered stomach always reacts on the teeth.

Most people are very incautious as to the temperature of the substances brought into contact with the teeth; and Dr. Combe goes so far as to warn his readers against allowing the cold night-air to come in contact with them after leaving hot rooms; and advises breathing through two or three folds of a silk handkerchief or through a woollen comforter. Results so much more serious than the toothach are produced by thoughtless exposure to the night-air, and sometimes, according to our own observation, fatal results after a single exposure, that it was with some degree of interest we recently heard of a contrivance called the *respirator;* a sort of veil of pierced metal, so contrived as to be worn over the mouth. It seemed to us that its utility would be great; but we fear that, like the safety-lamp of the miner, it would often be left at home when most wanted.

Like the generality of authors who write upon digestion and diet, Dr. Combe strives to convince his readers of the important preliminaries to good digestion which are designed to be effected, by the teeth and salivary glands, in the mouth. It unfortunately happens, however, that the custom of eating with extreme rapidity, commenced in very early life, proves too strong in most individuals to yield to any tardy conviction of the impropriety of neglecting due mastication. Indeed, whoever is animated by a desire to attend to this process will so often find himself left far behind in the race at dinner, and constituting part of so very small, however respectable a minority, as to be in much danger of being discouraged, and driven to swallow with the velocity of those around him.

Of all physiological facts, that of the nourishment of animal bodies by the food they take is the most familiar; yet no fact presents more matter for reflection. By what we call a law of the economy, the body grows for a certain number of years: bone, muscle, blood-vessel, membrane, parenchyma, and all the materials of the body, being formed out of the circulating blood, and the blood itself prepared in a manner that escapes all scrutiny. But, after a certain number of years, although food may be still taken in equal quantity, and all the materials of the body are formed with equal nicety, and deposited with equal exactness, each in its proper tissue and just place, *growth* proceeds no further. Still, for an appointed time, the daily elaboration of animal and vegetable substances produces all the required elements of the body, until other changes, the precursors of the body's death, announce its decay. Dr. Combe has given a clear and interesting account of the wonderful processes by which all this is effected, and of the organs that are employed in them, containing little or nothing new to medical readers, but much that is new and surprising to the general reader. The following particulars relative to the muscular coat of the stomach will, however, be new perhaps, even to some who are well acquainted with the facts generally known concerning digestion.

"The *second, middle,* or *muscular* coat consists of fleshy fibres, one layer of which, running longitudinally from the cardia to the pylorus, seems to be a continuation of the longitudinal muscular fibres of the gullet: another runs in a circular direction, embracing, as it were, the stomach from one curvature to the other, and constituting what are called the transverse fibres. A third and more internal layer of this coat, is spoken of by Sir Charles Bell as a continuation of the circular fibres of the gullet, which divide into two parcels, the one distributed over the left or larger end, and the other over the pyloric or narrower end. The uses of the *muscular* coat have, as we shall afterwards see, a direct reference to the special function of digestion. By the joint action of its longitudinal and circular fibres, the stomach is enabled to contract, and shorten its diameter in every direction, so as to adapt its capacity to the volume of its contents, while, by their successive action, or alternate contraction and relaxation, a kind of churning motion is produced, which contributes greatly to digestion by the motion which it imparts to the food, and the consequent exposure which it effects of every portion of it in its turn to the contact of the gastric juice. The force and rapidity of these muscular contractions are modified by the more or less stimulant nature of the food, the state of health, exercise, and other circumstances; but, according to Dr. Beaumont, the ordinary direction in which they take place, and the course which they impart to the food, are as follows. The alimentary *bolus* or morsel, on entering the cardiac orifice, turns to the left, follows the line of the great curvature of the stomach towards the pylorus, returns in the line of the smaller curvature, makes its appearance again at the cardia, and then descends as before to the great curvature, to undergo similar revolutions till digestion be completed. Each revolution occupies about from one to three minutes, and its rapidity increases as chymification advances." (P. 68.)

After speaking of the different functions of the nerves of the stomach, Dr. Combe devotes a considerable number of pages to the subject of the gastric juice. The strangeness of the case of Alexis St. Martin, related by Dr. Beaumont, of the United States, has apparently made a strong, and, we could almost say, an undue impression on Dr. Combe's mind; for the results are not many or even altogether novel, whilst even the most candid reader views the marvellous narration, however unjustly, with a slight degree of incredulity. The subject of the case, a young Canadian, was wounded by the discharge of a musket shot; the whole charge of powder and duckshot being received into the left side, at the distance of only one yard from the muzzle of the gun. The integuments and muscles were blown away, "to the size of a man's hand," the anterior half of the sixth rib was carried away, the fifth rib fractured; the lower portion of the left lobe of the lungs was lacerated, as also the diaphragm; and the stomach was perforated. A long and tedious process preceded recovery; and, a year after the accident, the injured parts were all healed, except the stomach, the perforation of which remained, and was two and a half inches in circumference. For some months longer, the food could only be retained by a compress and bandage being worn; but then "a small fold or doubling of the villous coat began to appear, which gradually increased till it filled the aperture, and acted as *a valve,* so as completely to prevent any efflux from within, but to admit of being easily pushed back by the finger from without." Two years after this period, Dr. Beaumont began his observations and experiments on this case, continuing them for four or five months; and four years afterward he renewed and completed them, having then induced the subject of them to reside near him for the purpose; which he appears to have done for about two years. The experiments were again renewed a year later, and continued for a few months. The subject of these experiments is still living, and in good health, although the perforation has now existed twelve years. It is situated about three inches to the left of the cardia, near the left superior termination of the great curvature. The means of observation were much facilitated by the circumstance that, when the stomach was nearly empty, it was practicable, by artificial distention, to examine its cavity to the depth of five or six inches. When the man had slept for a few hours on the left side, the protruded portion became larger, spreading over the integuments to the extent of five or six inches in circumference, and plainly exhibiting the mucous coat. The opportunity thus afforded for observation was certainly unusually advantageous; but the results appear to us to be less important than they have seemed generally to be considered. We do not, for example, recognize anything novel in the assurance that the gastric juice is not secreted in the intervals of digestion, and does not accumulate to be ready for acting on the next meal; nor in the fact, which might fairly be concluded from phænomena before known, that the villous coat of the stomach, when the individual is in a feverish state, becomes red and dry, or pale and moist, and loses its smooth and healthy appearance. Nor does the fact present itself to us at all in the light of a discovery, that in the same feverish condition no gastric juice is secreted, even when food is taken. To *see* such things is indeed curious, but the knowledge of them was previously established by symptoms during life, and by appearances after death. We accept with gratitude every kind of confirmatory evidence; but we object to exaggerating the importance even of such evidence, when drawn from an example in which it is difficult to admit the perfect integrity of the functions of the organ in question. There is sufficient novelty in Dr. Beaumont's method of procuring the gastric juice in this case to justify our quoting it, although the results are still, we think, of trivial importance.

"On examining the surface of the villous coat with a magnifying glass, he perceived an immediate change of appearance ensue whenever any aliment was brought into contact with it. The action of the neighbouring blood-vessels was instantly increased, and their branches dilated so as to admit the red blood much more freely than before. The colour of the membrane consequently changed from a pale pink to a deeper red, the vermicular or *worm-like* motions of the stomach became excited, and innumerable minute lucid points and very fine nervous and vascular papillæ could be seen arising from the villous coat, from which distilled a pure, colourless, and slightly viscid fluid, which collected in drops on the very points of the papillæ, and trickled down the sides of the stomach till it mingled with the food. This afterwards proved to be the secretion peculiar to that organ, or, in other words, the true *gastric juice*; the mucous fluid secreted by the follicles, which some have mistaken for it, is not only more viscid, but wants altogether the acid character by which it is generally distinguished. Pursuing his experiments, Dr. Beaumont then found that the contact not only of food but of any mechanical irritant, such as the bulb of a thermometer, or other indigestible body, invariably gave rise to the exudation of the gastric fluid from these vascular papillæ; but that, in the latter cases, the secretion always ceased in a short time, as soon apparently as the organ could ascertain that the foreign body was one over which the gastric juice had no power. But the small quantity obtainable in this way is perhaps more pure and free from admixture, and therefore better adapted for examination, than any which can be procured under any other circumstances." (P. 89.)

In the imposing list of "Inferences from Dr. Beaumont's Experiments and Observations," (p. 140,) and which contains no less than forty-five such alleged inferences, the medical reader will be surprised to find scarcely twelve that contain any novelty; and of these the greater part, if not all, appear to us to be of a very doubtful nature. If we admit amongst the least doubtful the inferences that the natural temperature of the stomach is about 100° Fahrenheit, and not elevated by the ingestion of food, but elevated by exercise, and depressed by sleep and rest in a recumbent position; and that gentle exercise facilitates the digestion of food, (and we admit even these with some abatements;) we are not at all convinced that hunger is the effect of distention of the vessels that secrete the gastric juice; nor that mastication and insalivation in no way affect the digestion of food; nor that the action of the gastric juice is facilitated by the motions of the stomach; nor that such motions produce a *churning* of the stomach's contents. And, as regards the rest of the inferences, about thirty in number, they seem to us to relate to facts very commonly known and very generally admitted before Dr. Beaumont began his experiments. We admire the patience and zeal of the experimenter, but we protest against being carried away by the novelty of the experiments to declare every inference confirmed by them an actual discovery. For example, the fact which seems to be brought forward with particular claims of this kind, that liquid food is immediately absorbed in the stomach, and disappears, was published by Magendie at least eighteen years ago. We cannot but think, also, that by far too much is assumed by Dr. Combe as the result of the muscular contractions of the stomach during digestion. That strong soups, and other forms of concentrated nourishment, are not really nutritious, and not in fact well digested, is, we believe, well established; but this circumstance may be accounted for without having recourse to the supposed difficulty with which the stomach contracts on such food; neither do we think that the statements of Dr. Philip respecting the order in which the food is digested, as observed in healthy animals, can be considered as put aside by a set of experiments on a man whose condition we should have some difficulty in admitting to be completely healthy at any period during the performance of the experiments of Dr. Beaumont. Altogether, we should have been better pleased with a more general account of the known facts respecting digestion, than we are with the devotion of so large a space in a popular work to the notice of experiments which have not yet passed the ordeal of professional examination.

The transmutation of all the varieties of food partaken of by man into chyme in the stomach, into chyle in the intestines, and into blood in the lungs,—the blood itself being a most complex fluid, from which all other fluids and all the solids of the body are formed,—comprehends so many extraordinary particulars, that the most minute observations yet made, and the explanations yet given respecting parts of the process, even of such as, resting on chemical principles, seem the most to be depended upon, leave us in a state of ignorance the most humiliating and profound. Yet it would seem as if the direction now taken by physiological enquirers, through the science of animal chemistry, promised by slow degrees to unveil some portions of the mystery, and, although still leaving the great principle of all the operations unknown, would revolutionize the whole of practical medicine; accounting for what was before empirical, and giving stability to therapeutics. But, before that great end is accomplished, many erroneous chemical theories will rise and fall, and many professed improvements in medicine will flourish for awhile, and then sink into decay. The range of chemical medicine is certainly yet very limited; and, what is still more discouraging, the facts ascertained concerning digestion have not been so fertile in good dietetic principles as might have been expected. The chyme, an acidified mass, passes from the stomach into the duodenum; and there the bile and pancreatic juice are added, and the chyme is separated into what is excrementitious and what is called chyle; and over this most important process we can exert little control, and of how it is completed we may be said to be very scantily informed. The portion of the process performed in the duodenum, and even in the long folds of the small intestines; the exact use of the caput cœcum of the colon, and of the expanded colon itself, are far too little known to warrant the confident opinions so frequently expressed concerning them. And, in the mean time, one cannot reflect without astonishment on the common and capricious employment of alkalies and of acids in medicine; so often prescribed with advantage where it would, we suspect, puzzle the practitioner exceedingly to account for his own success.

In the concluding chapter of the first part of his work, Dr. Combe gives a succinct account of the little that is known on the subject of Chylification; to which we think, however, that Dr. Prout's observations, detailed in his Bridgewater Treatise, might with propriety have been added. But it is necessary to take into consideration that the descriptive and physiological chapters of Dr. Combe's book are but introductory to those in which he endeavours to enlighten general readers on the important point of Dietetics, viewed in relation to the laws of digestion; and perhaps the work would really have been more useful, if the physiological or introductory part had been more condensed. Notwithstanding the clearness of Dr. Combe's style, we fear there is more said in that division of the work than general readers will readily comprehend or usefully retain.

The Second Part of the work is full of interest, and quite equal to the best portions of Dr. Combe's former publication. It relates to matters the very titles of which show that they concern all readers. The times of eating, the quantity and kinds of food, the conditions to be observed before and after eating, and the subject of drinks, are things of daily and serious import to all reflecting and to all unreflecting persons; and the pages devoted to these topics will be doubtlessly carefully studied by people of all ranks and occupations.

With that very considerable portion of mankind of which the happiness mainly depends on corporeal enjoyment, there is apparent a perpetual desire to participate in the happiness so bountifully imparted to some of the most inoffensive of the lower animals; and which arises from continual eating. We hope not to be reproached with the levity of satire if we declare that, to our observation, a most lamentable majority of our country friends awake in the morning to no nobler desire than that of a hearty breakfast; and that, when this meal is happily concluded, their thoughts seem to turn with diurnal regularity towards luncheon, from which, by gentle transition, they pass into more serious arrangements as to dinner; closing the thankful day with solid supper, in defiance of all the practitioners upon earth. In the finer season, even the intervals between more satisfactory meals are often much filled up with forbidden fruits in the garden; and tea and bread and butter are not rigorously taken into our calculation. We have sometimes blamed the practitioner for being so unprepared with dietetic rules; but there are emergencies which baffle the wisest. How often does it happen that his patience is tried, on quitting the room of a patient in a state of disease attended by excitement and feverishness, by his being asked what the invalid must eat, and whether something nourishing ought not to be taken every hour or two. There are numerous families accustomed to so indulgent a sway as to render all the members averse to regulations which enforce any three hours in the whole day being passed wholly without food. People of the class accustomed to this plan commonly eat five times a day. People of fashion, scorning such a laborious system of nutrition, make it their boast that they only eat twice a day. Thus, whilst no small division of the agricultural population resist the langours of a rural life by eating at four or five in the morning, and again at seven or eight, and again at eleven or twelve, and again at four or five in the afternoon, and again at eight or nine in the evening; the more refined inhabitants of the metropolis, and the aristocracy of aspiring provincial towns, take much credit to themselves for not breakfasting until the middle of the day, (that breakfast being a very substantial dinner,) and for not dining before night, (that dinner being a consolidation of dinner and supper.) Thus it would seem that in eating and drinking, as in morals, men ever miss the golden mean. As some grovel in low and hopeless vice, and others subtilize themselves into impracticable virtue; so some torment the stomach by gross and perpetual labour, and others provoke it by alternations of fulness and fasting; and indigestion, with more than poetical justice, seizes upon them all, and consigns them to the salutary discipline of medicine.

It is the aim of Dr. Combe's excellent book to relieve mankind from these evils,—the evils of indigestion, with all its numerous consequences, at which none laugh but those who never felt any of them, and from which it is sufficiently discreditable to human reason that it has not sooner emancipated all persons of tolerable education and healthy understanding. Of all the useful arts, the art of happiness seems to have yet attracted the smallest share of attention; and of that art a very principal element is surely the preservation of mental and bodily comfort, neither of which are compatible with disorder in the first passages, with commotion in the stomach, and rebellion in the bowels.

We are acquainted with individuals for whose opinions we entertain much respect, whose imagination ever pictures to them some region secure from the formal invasion of a daily dinner, and who silence their friends, if they do not convince them, by earnest invectives against regulating meals by measurements of time, and disregarding the promptings of unerring nature, which tell us to eat when we are hungry, and to drink when we are thirsty. We do not see that Dr. Combe sanctions these philosophical sophistries. He truly observes, that there is an evident adaptation of the human economy to the performance of periodical operations, and that stated times for eating may really be observed without any departure from rationality of conduct. In truth, the argument of the philosophers might be as properly extended to sleep; and if, on the one hand, a family might escape the torment of a long dinner by a readier access to the temporary succours of the pantry or the tray, so, on the other, might they be delivered from the slavery of periodical repose, and indulge in sleep in many stolen hours of day, regardless of place as well as of time. But this would evidently be too inconvenient to find general encouragement in regular families; and the same may be said of the plan of independent eating; whilst neither, in all probability, would prove more conducive to health than the present mechanical observances. We may say, indeed, with Dr. Combe, that individuals may suffer a little from existing arrangements, but the vast majority are undoubtedly benefited.

"As a general rule," says Dr. Combe, "five or six hours should elapse between one meal and another—longer if the mode of life be indolent, shorter if very active. Digestion occupies from three to four or five hours, and the stomach requires an interval of rest after the process is finished, to enable it to recover its tone, before it can again enter upon the vigorous performance of its function. Appetite, accordingly, does not begin to shew itself till some time after the stomach has been empty, and if food be taken before it has recovered its tone, the secretion of gastric juice and the contracting of its muscular fibres are alike imperfect, and digestion consequently becomes impaired.

"The interval between each meal ought to be longer or shorter in proportion to the quantity eaten, and to the more or less active habits of the individual; and it would be absurd to fix the same standard for all. A strong labouring man, whose system is subjected to great waste from being engaged all day in hard work, will require not only more frequent but more copious meals than an indolent and sedentary man; and those who eat very little will require to eat at shorter intervals than those whose meals are heavy. An invalid on restricted diet may thus require to eat every four hours, where formerly, with a more copious diet, once in six hours was sufficient." (P. 182.)

If many of those whose lot has fallen in lands flowing with plenty assimilate themselves with animals whose harmless lives are chiefly passed in the mastication of food; so, on the other hand, those who, in uncivilized countries, depend for a dinner on the uncertain supplies of the chace, are habituated to take sufficient at one meal to fortify themselves against the chances of passing at least twenty-four hours without another; and habit probably renders this system as favorable to the force of digestion in the Indian as the committee of the Zoological Society report it to be to the health of the wild animals under their protection in the Regent's Park. Dr. Combe relates that he once travelled with a monk of La Trappe for three days, who observed this plan; and the poor monk ate once a day, to the very brink of apoplexy.

Every practitioner knows with what reluctance sluggish self-indulgent people are brought to walk into the open air before breakfast. Some have the excuse of its disagreeing with them; and there are few who can bear much exertion before any food is taken. We are, however, of opinion that exposure to the air, if only for a quarter of an hour, in all tolerable weather, before breakfast, has a great tendency to strengthen the stomach, and to promote the action of the bowels; so as to be well worth the attention of medical advisers in cases of habitual costiveness. In marshy situations even this should be done with caution; and long and early parades have, in such circumstances, seemed most prejudicial to the health of soldiers. If, as is alleged, there is in such circumstances a greater susceptibility of infection and other poisonous influences, we should ascribe it as much to the want of tone and resistance in individuals so exposed, after fasting several hours, as to the higher power of the function of absorption in the morning, on which Dr. Combe lays so much stress. The effect, whatever the cause, should be remembered by those in attendance on patients affected with fever, and a little coffee or other refreshment should be allowed to all persons engaged in such perilous duties.

The commercial part of the community may read with great advantage Dr. Combe's remarks on the pernicious habit, previously and strongly pointed out by Dr. Paris, of taking exercise after many hours' confinement to business, and while yet they have not dined. Upon the whole, we should agree with Dr. Combe, that those who breakfast at nine should dine at two. But many are engaged in business or in pleasure, and have not time to dine at two, or imagine that they have not, because they consider that dinner ought to be a very substantial meal; and, after such a meal, men are more inclined to sleep than to work. We must therefore compound with them in these circumstances, and delude them, as they love to be deluded, with names. Call a light dinner *luncheon,* and the man of business finds time for it, and the man of fashion no longer disdains it. Call an early supper a late *dinner,* and the merchant and the senator will sup contentedly, but by far too luxuriously, at the time when peasants sup. From the excess of this last meal, to which no exercise succeeds, the voice of the physician, charm he ever so wisely, cannot warn them, until auxiliaries rise up in the shape of gout or gravel, and rich men grow virtuous in spite of themselves.

At the same time, Dr. Combe very justly observes that those who sit up a great part of the night require food at later hours; and every one must have experienced the welcome refreshment of supper at a fashionable party. Sometimes, indeed, the exhibition of hunger, even in refined circles, towards two o'clock in the morning, is irrepressible; and, whilst it furnishes a scene for the writer of a novel, affords instruction, as every other scene does, to the physiologist.

The refinements of dietetic writers have often excited a suspicion, which daily observation has a tendency to confirm, that, after all, it is the *quantity* of food taken, or of particular kinds of food, that is the most common cause of the inconveniences ascribed by different authorities to different articles of diet. It would be endless to recount the extravagances of some of the foreign writers concerning the effect even of some of the commonest articles of subsistence. According to one, the prevalence of consumption in England is to be ascribed to the habitual use of butter; but of such follies any notice is, we trust, superfluous. We place no very great reliance on the rule of leaving off eating with an appetite; and still less on Dr. Beaumont's refined distinction between leaving off with "satiety," which he pronounces pernicious, and with "perfect satisfaction, ease, and quiescence of body and mind," which he declares to be signals for desisting from eating. We profess an incapacity quite clearly to discriminate between *enough* and *satiety.* We know that our lexicographers define satiety to be *more* than enough; but we distrust the memory of people who are at dinner concerning these nice points. The best rules are, perhaps, to eat slowly, and not to partake of too great a variety, and not to drink much or anything at meals. The last rule cannot always be rigidly enforced; but we believe its virtue to consist in the smaller quantity of food to which it limits the desire of the person dining. By observing these rules, few people would err greatly on the side of quantity; whereas, rapid swallowing, varieties of food, and large supplies of fluid stimulants, inevitably lead to the overloading of the stomach.

Dr. Combe's usual good sense does not desert him on these important matters. He abjures all universal systems of diet, either as regards quality or quantity, observing that, in different ages, after different degrees of exertion, and in different ranks and circumstances, different rules must be observed. Infants require frequent supplies of food; but every day shows us the bad effect of the excessive feeding to which these helpless creatures are subjected. The Esquimaux system is indeed literally practised upon them; they are crammed until their very mouths can hold no more. Hence ensue vomitings and what are called bilious attacks in very young children, for which they are submitted to calomel and various purgatives, in default of any correction of the bad habits of the nurse or the mother. All the time, too, the nurse is carefully fed, and expected to drink ale or porter, or even gin, to increase the quantity of milk. We are strongly impressed with the belief that it is this mismanagement of their own diet, rather than affording milk to an infant, which causes the mother so frequently to find her constitution impaired by nursing. There are doubtless cases in which mothers, from absolute debility, are unfit nurses; but we should be disposed to maintain that no healthy woman requires an unusually full diet to enable her to secrete sufficient milk for the nourishment of her child; and that, when this cannot be done upon good diet, without the unusual indulgence of strong fermented liquors, or of ardent spirits, it would be much better, both for the mother and the child, that the latter should be brought up by hand.

We do not think that practitioners are fully aware of the silly and hurtful excess in which women in very respectable classes of life indulge themselves, on the plea of being nurses. Much of this is occasioned by the prevalent mistake, so well dwelt upon by Dr. Combe, that every expression of uneasiness on the part of an infant is an indication of hunger, and that, whenever it opens its mouth, the mouth ought to be filled with food. It would be well if all mothers and nurses would read and remember his observations on the feeding of newly-born infants. We have indeed often witnessed with sorrow the eagerness displayed to load and to unload the stomach and bowels of a poor child, which has only within an hour seen the light. The contest seems to be, who shall first introduce it to the sufferings of colic or the miseries of medicine. It would be ludicrous, if it were not really horrible, to behold an able-bodied nurse giving a helpless infant a welcome to this world of trial in the shape of a dose of castor-oil. With this system of feeding and physicking, so far from wondering at the mortality among infants, one is almost inclined to regard it as a merciful provision against the continuance of such ignorant tyranny.

Passing over many valuable remarks on the quantity of food adapted to the years of growth, and to subsequent periods of life, we turn from the effects of over-feeding to the more pitiable effects of a want of proper nourishment; effects too little reflected upon even by medical men. We can add nothing to the force of the following observations: they ought never to be forgotten, and contain truths to which legislators are only now beginning to pay a tardy attention.

"If over-feeding be the prevailing error among the middle and higher classes of the community, the opposite condition is as unquestionably that of a large proportion of the labouring poor. Pressed upon all sides by the powerful competition both of constantly improving machinery and of a superabundant population, the manual labourer is impelled to undergo an amount of ever-recurring bodily exertion which far exceeds the natural powers of his constitution, even when supported by the fullest supply of nourishment; and when, as often happens along with this excess of labour, his food, from inadequate wages, the size of his family, or his own injudicious management, is defective in quantity or in quality, the consequences to his health and happiness are disastrous in the highest degree.

"To those who have never reflected on the subject, it may seem like exaggeration to say that, as a general fact, at least nine-tenths of the lower orders suffer physically, morally, and intellectually, from being overworked and underfed; and yet I am convinced that the more the subject shall be investigated, the more deeply shall we become impressed with the truth and importance of the statement. It is true that very few persons die from actual want of food; but it is not less certain that thousands upon thousands are annually cut off, whose lives have been greatly shortened by excess of labour and deficiency of nourishment. It is a rare thing for a hard working artizan to arrive at a good old age. They almost all become prematurely old, and die off long before the natural term of life. It is in this way that, as remarked by Dr. Southwood Smith, the mortality of a country may be considered as an accurate indication of the misery of its inhabitants. According to Villermé, the rate of mortality among the poor is sometimes double that among the rich. Thus it is found, he says, that in a poor district in France one hundred die, while in a rich department only fifty are carried off; and that, on taking into account the whole population of France, a child born to parents in easy circumstances has the chance of living forty-two and a-half years, while one born of poor parents can look for no more than thirty.

"These are striking facts, and their truth is confirmed by every day's experience in Britain as well as in France. Many causes concur to produce this melancholy result, but among the principal is unquestionably the disproportion so generally existing between toil and nutrition. In the army the operation of the same principle has long been recognised in the inferior strength and health of the privates compared with the officers.

The officers, being better fed, better clothed, and better lodged than the common soldiers, bear up successfully against fatigue and temporary privations by which the latter are overwhelmed. During epidemics, too, the poor, from their impaired stamina, almost invariably become victims in a proportion far exceeding that of the more wealthy classes. This is, no doubt, partly owing to their greater intemperance and want of cleanliness; but even these vices often derive their origin from the same root —the want of adequate repose and comfortable sustenance.

"The bad consequences of defective nourishment are not confined in their operation to the bodily constitution of the labouring poor. *Their minds also are deteriorated.* The pressure of poverty is unfavorable to the growth of refinement and morality, and crime and turbulence are never so much to be dreaded as during times of scarcity and manufacturing or agricultural distress. Bodily health, satisfied appetite, and peace of mind, are great promoters of individual morality and public tranquillity; and whenever these are encroached upon in any great class of the community, discontent and crime are sure to follow. In legislation this principle is seldom attended to, and laws are consequently enacted merely for the suppression of the result, while the source from which it springs is left altogether unnoticed and in the fullest activity.

"Among the poorer classes, the children as well as the parents suffer much both physically and morally from insufficient food. Their diet, being chiefly of a vegetable nature, and consisting of porridge, potatoes, and soups, with very little butcher-meat, proves far from adequate to carry on vigorous growth in the one, or repair waste in the other; hence arise in the young an imperfect development of the bodily organization, a corresponding deficiency of mental power, and a diminished capability of resisting the causes of disease. In workhouses and other charitable institutions, ample evidence of these deficiencies obtrudes itself upon our notice, in the weak and stunted forms and very moderate capacities of the children. Under an impoverished diet, indeed, the moral and intellectual capacity is deteriorated as certainly as the bodily; and a full exposition of this fact, and the principles on which it is founded, would be a great public benefit." (P. 234.)

In the chapter on the Kinds of Food, Dr. Combe recurs to the subject of infants and nurses; and, if his book contained no other good advice, this part of it alone ought to recommend it to every family. There is a singular disregard of certain tolerably plain indications of nature in much that relates to infants and children. Infants delight in nothing so much, for example, as lying on the nurse's lap divested of clothing. A purer picture of happiness cannot be conceived than that presented by an infant in this situation; yet it is condemned to complicated clothing, by which its skin and limbs are compressed and irritated. The child of older growth cries in the cold, and is yet condemned to long walks in cold air, and sometimes to a cold bath daily. But the errors as respect the food proper for infants are yet more remarkable. Generally they are fed on food so much thicker than the milk of the mother, that their digestive organs are seriously discomposed by it; and they are often kept on the mother's milk until long after teeth have appeared, which nature has furnished for the purpose of biting and masticating. To some, animal food is given before the teeth have well appeared; and to some it is denied long after thinner aliment has evidently ceased to afford nourishment. Mothers of the poorer class are continually applying at dispensaries for medicine for children of twelve or eighteen months old, whose health is merely deranged by their not being weaned; and we fear that Dr. Loudon's late ingenious recommendation to limit the population to the sustenance of a country may be opposed by irresistible arguments, drawn from the effects of long-continued nursing on the mother. Another circumstance to be taken into consideration, too, is that mothers often prolong the period of nursing with the exact intention for which Dr. Loudon recommends it. They wish to limit the population of their own cottage to the sustenance. But, in numerous cases the plan does not succeed; and the uterine system, after a certain period allotted to nursing, claims an ascendency over the mammary; a fact of considerable physiological interest, and on which it would be easy, and not without use, to collect precise statistical details in lying-in institutions.

These observations will shew that we do not quite agree with Dr. Combe when he says, that, "in general, weaning takes place too early," (p. 248;) but his directions both as respects the food of infants and of nurses are quite unexceptionable. If he dwells on the mischief which ensues from giving too much food to infants, he does not forget the necessity of a copious supply of good food to older children, and he advocates neither excess nor too sparing a diet. We would here beg to repeat that the best rules appear to us to be, to give growing and healthy children good plain food at regular hours, and to prevent their eating too fast, or drinking too much with their meals, or taking any stimulants; and, in that case, they will seldom eat too much. They require a full measure of food; but, if they are allowed to be active, and much in the open air, their strength, their clear complexion, their cheerfulness, and their balmy sleep at night, will shew that the system is not overloaded or injured. Often as the subject spoken of in the following extract has been of late presented to the attention of the medical and even of the general reader, we cannot refrain from again urging continual attention to it. On these points, medical men are the teachers of the community; a proud privilege, and which should not be neglected, for the teaching relates to health and to happiness.

"The prevalent and pernicious custom of tasking the minds and confining the bodies of children for hours in succession at home and in schools, at a time of life when the growth of the body and the welfare of the system require constant and playful exercise in the open air, and perfect freedom from care and excitement of mind, is the fruitful source of much future bad health, and is eminently calculated to defeat the object aimed at by parents, namely, the mental excellence of the child. The premature exertion of intellect to which it is stimulated by the constant excitement of emulation and vanity, far from strengthening, tends to impair the health and tone of the brain, and of all the organs depending on it; and hence we rarely perceive the genius of the school manifesting in future years any of the superiority which attracted attention in early life; but we find them, on the contrary, either sunk below mediocrity, or dragging out a painful existence, the victim of indigestion and melancholy. On the other hand, some of the most distinguished men who ever lived were in childhood only remarkable for health, idleness, and apparent stupidity. The illustrious Newton was, by his own account, an idle and inattentive boy, and 'very low in the school,' till he reached twelve years of age; and the young Napoleon himself is described as 'having good health, and being in other respects like other boys.' Adam Clarke was considered 'a grievous dunce' when a boy, and was seldom praised by his father except for his ability *in rolling large stones*, which his robust frame and good health enabled him to do. Shakspeare, Gibbon, Byron, Scott, and Davy, were in like manner undistinguished by precocious genius, and were fortunately allowed to indulge freely in those wholesome bodily exercises, and that freedom of mind, which contributed so much to their future excellence." (P. 254.)

Dr. Combe frequently refers to Dr. Clark's admirable observations on diet and regimen as a means of preventing scrofula and consumption; ends so important, that we cannot but hope some good has already been effected by Dr. Clark's remarks. In families which have already suffered the deep affliction of seeing one or two of the younger members sinking under pulmonary consumption, no words could add to the awful interest attached to any directions by which a chance is afforded that the survivors may be spared.

On the subjects of particular articles of diet, and on the conditions to be observed before and after eating, we do not observe much in the pages of Dr. Combe which has not previously and for some time been familiar to all medical readers. We need scarcely say, that we attach no great importance to those of Dr. Beaumont's experiments which demonstrate the superior digestibility of raw cabbage; although we cannot but offer our congratulations to Dr. Lambe on such a confirmation of opinions which that very respectable physician is well known to have consistently maintained for many years, both in theory and practice, although with the effect of making very few converts.

In a chapter on the subject of Drinks, Dr. Combe refers to the experiments of Dupuytren and Orfila, in support of the opinion that thirst is very much dependent on the quantity of fluid circulating in the vessels. Dupuytren succeeded in allaying the thirst of animals by injecting milk, whey, water, and other fluids into the veins; and Orfila frequently, in the same manner, allayed the excessive thirst of animals to which he had administered poison, and which were incapacitated from drinking by the œsophagus being tied; and he found that, the more the abstinence from liquids was prolonged, the more the blood was deprived of its watery portions. Dr. Combe, however, guards his readers from the error of drinking fluids to excess, although in obedience to what may seem a natural want of them in the system. We have already said, with reference to a well-known precept of Mr. Abernethy, that, by drinking little at dinner, less solid food will also be taken; and this we believe to be the best effect of observance of a rule which has puzzled many who have obeyed it.

In one of Dr. Beaumont's experiments, a gill of water, at the temperature of 55° Fahr., was received into the stomach, the temperature of that organ at the time being 99°. The water immediately diffused itself over the interior surface of the stomach, reducing the temperature to 70°. After a few minutes, the temperature of the stomach began to rise again; but it did not regain its former level of 99° until half an hour had elapsed. If this effect of so small a quantity of water throws some light, as Dr. Combe observes, on the danger of taking large draughts of water at a lower temperature, it also, we would add, illustrates the action of refrigerants, about which many practitioners are very sceptical. The fact may be also recommended to the reflection of ladies and gentlemen who so fearlessly devour ice immediately after dinner, coupled with the fact supposed to be established by Dr. Beaumont, that, for good digestion, the temperature of the stomach should be about 100°. The warmth or the coldness of fluids swallowed should of course also be attended to; the point of wisdom being that any extreme is hurtful, and, if habitual, more hurtful.

If much fluid of any kind drunk during a meal has the effect not only of causing more food to be taken than would otherwise be required, and if it also, as is generally admitted, dilutes and impairs the efficacy of the gastric juice, it may readily be concluded that, if the fluid taken be itself of a kind requiring the digestive process to be exercised upon it, or if it be of a stimulating quality, it should be partaken of with caution. Those whose practice makes them acquainted with the maladies of large drinkers of ale or porter, are aware that the number and severity of the disorders induced by this kind of excess are not less than what are incurred by persons who indulge much in wine, or even in ardent spirits. The affections induced by an imprudent indulgence in these various stimulants are not the same, but they all indicate an impairment of health, either in the form of dyspepsia, or of evident vascular or nervous derangement. Yet there is much reason to believe that a total abstinence from stimulating drinks is not compatible with the health of many individuals in the circumstances of social and active life. Ardent spirits should unquestionably never be used but as an occasional cordial or needful medicine; and, of the importance of habitual moderation in the use of wine and of malt liquors, no reasonable man can entertain a doubt. Every employment of these beyond the sensation of agreeable refreshment tends to impair the organs or parts of the system on which the particular stimulant chiefly acts. Youth, strong exercise, and an excellent constitution, may postpone the bad effects of stimulants even in those who indulge with little restraint in their use; but, when middle age is attained, the days of retribution seldom fail to commence.

Dr. Combe's opinions on these subjects are such as we entirely concur in. To most children we believe the use of wine or of any other stimulating drink to be positively injurious, and to young persons it is unnecessary. In active life, some of them may be habitually and moderately partaken of with advantage, and also in life's decline. They have been permitted to man for his use, and for his benefit; and, if his reason is employed to direct that use of them, as of other blessings, the benefit may be enjoyed without any penalty.

It is too well known that stimulants are often resorted to even by persons of cultivated understanding, to cheer them in melancholy circumstances. Of course, by injuring the health, they add to the depression. Partaken of in great moderation, even in such circumstances, they are doubtless sometimes beneficial, rousing the natural energies of the system without exhausting them, and allaying, for a time, the irritability of the mind. By the poor, spirits are taken as their solace and only enjoyment. Those who so laudably wish to wean them from such pernicious and destructive consolation should remember, that the best means of doing so consist in bettering their condition, improving their prospects, and enlarging their mental resources. Without such care, oaths, like precepts, will soon be forgotten.

A short and judicious chapter on the Regulation of the Bowels concludes the work, a chapter rendered necessary by the too general practice of seeking in the daily use of purgatives a substitute for a better ordered diet and daily walking exercise. In this respect modern practice has but too much coincided with popular errors. Nothing is so rare as to meet with a prescription in which no cathartic substances are included. We once inspected a file of prescriptions in the possession of a lady who had been affected with chronic diarrhœa for nearly two years, and found calomel or blue pill, generally combined with a purgative, in every one of them. Women of every degree, but particularly of the middle and higher classes, are accustomed to take daily aperients, neglecting attention to diet, and refusing to take any active exercise in the open air. To such an extent does this very foolish and hurtful plan prevail, especially in large towns, and above all in London, that a practitioner may do nearly the same amount of good by prescribing the discontinuance of medicine as by prescribing any to be taken.

Our notice of Dr. Combe's book sufficiently shews that we think it a useful production. It somewhat abounds in repetitions, and bears, in our opinion, other marks of the kind of expansion generally required in works which follow a successful one by the same author. The most valuable parts of it, considering the readers to whom it is addressed, might have been considerably condensed; or might even, we think, have been incorporated into the first publication of the kind of which Dr. Combe was the author. For those general readers, however, who will patiently peruse it, the Physiology of Digestion will be found no less really instructive than the admirable Physiology applied to Health and Education. As the author of each of these works, we certainly regard Dr. Combe as a benefactor to the reading public.

---

